# Commentary: Muscle Microbiopsy to Delineate Stem Cell Involvement in Young Patients: A Novel Approach for Children With Cerebral Palsy

**DOI:** 10.3389/fphys.2021.642366

**Published:** 2021-02-05

**Authors:** Richard L. Lieber, Andrea A. Domenighetti

**Affiliations:** ^1^Shirley Ryan AbilityLab, Chicago, IL, United States; ^2^Department of Physical Medicine and Rehabilitation, Northwestern University, Chicago, IL, United States; ^3^Hines VA Medical Center, Maywood, IL, United States

**Keywords:** cerebral palsy, skeletal muscle, satellite cell, cell cultural, stem cell

## Introduction

**Figure 1:**
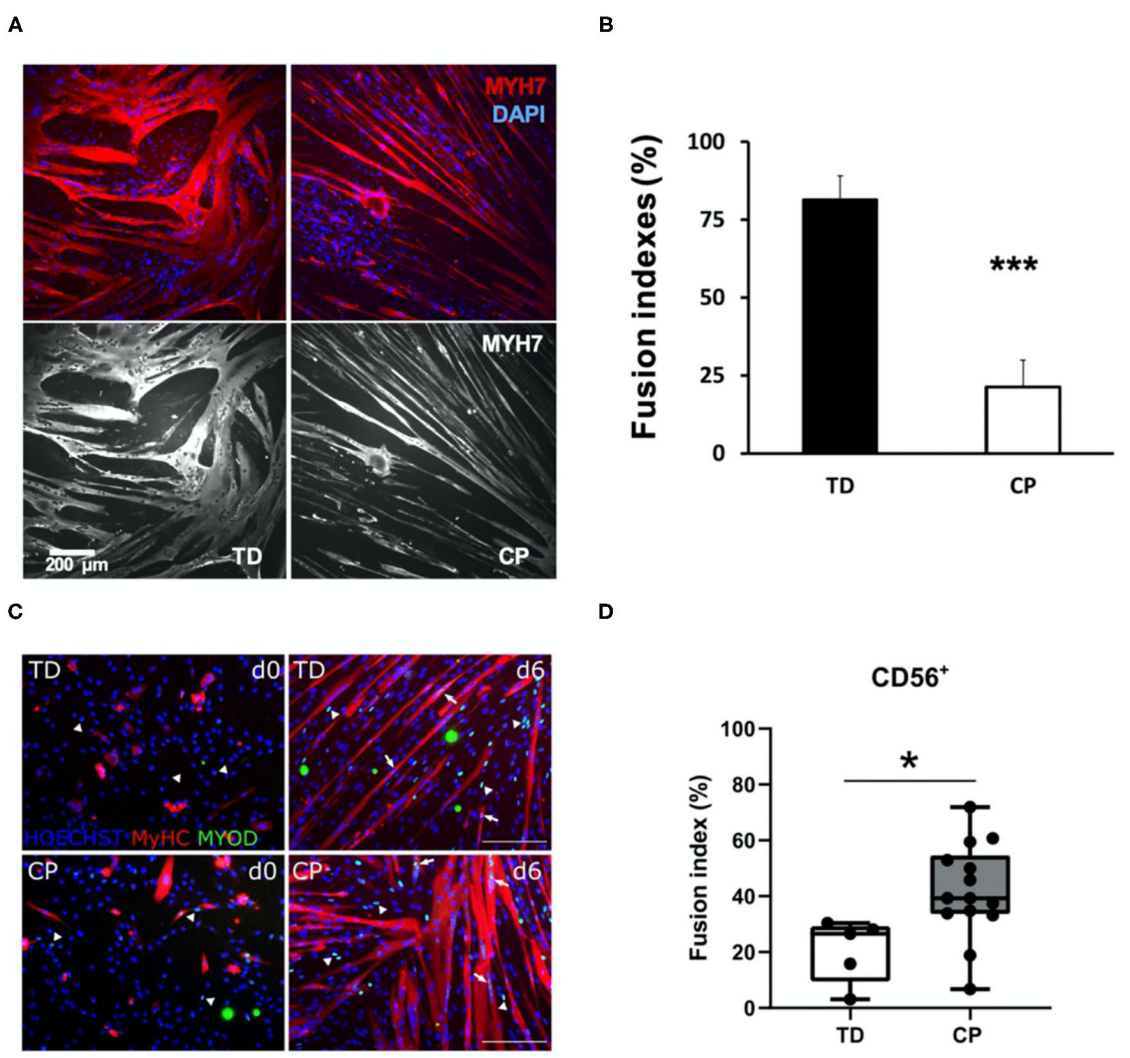
Comparison of immunohistochemical appearance and fusion indices between studies. **(A)** Myoblasts from CP and TD preparations were differentiated for 42 h and stained for slow myosin heavy chain (MYH7) and a nuclear stain (4-,6-diamidino-2-phenylindole, DAPI). CP myotubes appeared spindly, thin, and with fewer nuclei per myo- tube. Gray scale panels show MYH7 staining as a single channel. **(B)** Quantification of fusion index for CP and TD myoblasts. Quantification was performed after 42 h of differentiation; ***CP vs. TD, *P* < 0.001 (*n* = 8 per group). Figures from Domenighetti et al. (2018). **(C)** Representative immunofluorescence from satellite cell-derived progenitors of a TD child and a CP patient at days 0 and 6 of myogenic differentiation. MYOD+ nuclei (green) are highlighted by arrows when included into myotubes and by arrowheads if not yet fused; MyHC (red) and nuclei are counterstained by HOECHST (blue). Scale bar: 200 μm. **(D)** Fusion index (FI) values are represented by boxplots and dots represent individual subjects (TD: *n* = 5; CP: *n* = 14; *p < 0.05). Figures from Corvelyn et al. (2020).

We appreciate the opportunity to comment on the above-referenced article from our colleagues in Belgium. Our goals are (Domenighetti et al., 2018) to provide important background for this and related studies and (Lieber et al., 2003) to opine regarding apparent differences between this work and our previously published paper on the same topic (Domenighetti et al., 2018) (Figure 1).

To begin, we would like to state that we agree with the authors that the study of muscle growth and development (myogenesis) in cerebral palsy (CP) is an important and timely topic and that performing these studies (which requires direct access to human muscle tissue) is extremely challenging. We previously performed a number of these studies (Lieber et al., 2003; Smith et al., 2009, 2011, 2012, 2013; Dayanidhi et al., 2015; Domenighetti et al., 2018), including three relevant investigations on resident muscle stem cell homeostasis in contractured CP muscle (Smith et al., 2013; Dayanidhi et al., 2015; Domenighetti et al., 2018). Our studies were performed in children with spastic CP of an average age of 9, 11, and 13 years respectively, and with motor dysfunction rankings I–V of the Gross Motor Function Classification System (GMFCS). Some limitations of our studies included low sample sizes (*n* = 6–10 children per group) and age differences between groups in two of these studies, with children with CP being a few years younger than typically developing (TD) control children who donated muscle tissue during ACL reconstruction surgery (average age of ~14 years) (Smith et al., 2013; Domenighetti et al., 2018).

Corvelyn et al. (2020) are to be congratulated for circumventing some of these biases by developing a method to obtain a non-intraoperative but smaller (~10 mg) muscle biopsy by needle (Bergstrom, 1975), from less severely affected (GMFCS I–III) and generally younger children (average ~6 years of age). Unfortunately, their study lacked clarity regarding the actual muscle stem cell population being studied. This is based on the method chosen to extract myogenic cells from the very small biopsy and the need to expand and passage these cells multiple times before FACS analysis. As such, without further characterization of these cells, it is very difficult to interpret their results in light of muscle stem cell biology.

## Satellite Cell Homeostasis in Children With CP

While multiple resident and non-resident stem cells can support myogenesis, including fibroadipogenic stem cells (FAPs) (Joe et al., 2010), pericytes (Crisan et al., 2008), and other cell types (Brack and Rando, 2012), the actual primary self-renewing stem cell that proliferates, differentiates and fuses to produce a muscle fiber is the PAX7-expressing satellite cell (SC) (Schultz and McCormick, 1994; Brack and Rando, 2012; Yin et al., 2013). Muscle SCs are absolutely required for post-natal muscle development and growth (Cardasis and Cooper, 1975; White et al., 2010; Delhaas et al., 2013; Duddy et al., 2015; Gattazzo et al., 2020). When resident SC number or rates of myogenesis are decreased in a non-physiological manner during postnatal development, it leads to impaired muscle growth or recovery from contracture (Murach et al., 2017; Bachman et al., 2018; Chang et al., 2019; Dayanidhi et al., 2020). We and others showed that the PAX7-expressing SC pool is significantly reduced in contractured hamstrings and biceps of 3–18 years old children with CP (Smith et al., 2013; Dayanidhi et al., 2015; Von Walden et al., 2018). We also showed that myoblast progenitors derived from the same SCs (enzymatically extracted from contractured hamstring muscles and FACS-sorted as CD34^−^, CD45^−^, CD56^+^ cells) had decreased capacity to fuse and to differentiate into myotubes *in vitro* (Domenighetti et al., 2018). This result is in apparent contrast to what Corvelyn et al. observed with their mixed CD56^+^ cell populations (Figure 1).

## Different Experimental Methods to Isolate Myogenic Stem Cells

Based on their very small tissue sizes, Corvelyn et al. were forced to allow cells to grow out of the biopsy onto a culture dish for several days (see their Figure 1), and then expand them *in vitro* for several passages to obtain sufficient numbers of cells to sort into CD56^+^ and CD56^−^ populations by FACS. There are two major drawbacks with this approach: First, is lack of control over which cells (and in what percentages) will colonize the plate and continue to expand over several passages, leading to highly variable cultures. Second, without preplating (Yoshioka et al., 2020), growth of primary myogenic cultures over time will result in non-myogenic enriched populations of cells (Rando and Blau, 1994), while SCs will differentiate into myoblasts and rapidly lose their potential to self-renew and contribute to muscle fiber formation (Cosgrove et al., 2009). These limitations necessitated a longitudinal characterization of cell types at the time of plate colonization and then during amplification. While Corvelyn et al. confirmed presence of CD56^+^ and MYOD^+^ myogenic cells *in vitro* (their Supplementary Figure 3A), cell sorting should have included additional negative selection markers and a verification that a sampling of CD56^+^ sorted cells were also PAX7^+^. This was not done and the amplified cell type(s) are not known.

Thus, we believe that apparent biological differences between our two studies (Figure 1) are mainly caused by a lower-than-expected myogenic potential of cell cultures in Corvelyn et al. Modest upregulation of myosin heavy chain (MyHC) during 6 days of differentiation is indicative of this phenotype (see their Figure 3A). Furthermore, myogenic potential of their TD CD56^+^ cultures (~20% fusion index after 6 days of differentiation) is significantly lower than expected for human SC-derived myoblasts (60–80% fusion indices after 24–48 h of differentiation) (Fischer-Lougheed et al., 2001; Cerletti et al., 2006; Agley et al., 2017; Catteau et al., 2020). Thus, our provisional interpretation of their data is not that CP cells were more myogenic than TD but that, for unexplained reasons, the TD cells were grossly underperforming. It is also possible that muscle-specific differences in the differentiation ability of isolated myogenic progenitors could also have contributed for some of the differences observed between our studies. However, since both of these muscles have the same embryonic origin, we think this unlikely (Zammit, 2008).

## Discussion

Our goal in this commentary was to provide background insight into the complexity of performing such *in vitro* experiments from tissue extracted from young children, and the resulting difficulty interpretating results when the cellular identity is not clear. We congratulate the authors on completing a very difficult study and offer this critique in the spirit of improving all of our experiments and with the hope of uncovering new insights into etiology and treatment of cerebral palsy. Specifically, we believe that the optimization of techniques for SC isolation from small muscle biopsies of very young children (e.g., 0–3 years old) will significantly improve our understanding of early/developmental biological mechanisms that lead to motor and muscle impairments (including contracture development) in CP. We welcome continued development of these techniques of SC isolation.

## Author Contributions

RL and AD conceived, wrote, edited, and approved this commentary. Both authors contributed to the article and approved the submitted version.

## Conflict of Interest

The authors declare that the research was conducted in the absence of any commercial or financial relationships that could be construed as a potential conflict of interest.
